# Cooperative behaviour and phenotype plasticity evolve during melanoma progression

**DOI:** 10.1111/pcmr.12873

**Published:** 2020-03-20

**Authors:** Emily J. Rowling, Zsofia Miskolczi, Raghavendar Nagaraju, Daniel J. Wilcock, Ping Wang, Brian Telfer, Yaoyong Li, Irene Lasheras-Otero, Marta Redondo-Muñoz, Andrew D. Sharrocks, Imanol Arozarena, Claudia Wellbrock

**Affiliations:** ^1^ Manchester Cancer Research Centre Division of Cancer Sciences Faculty of Biology, Medicine and Health University of Manchester Manchester UK; ^2^ Bioinformatics Core Facility Faculty of Biology, Medicine and Health University of Manchester Manchester UK; ^3^ Division of Pharmacy and Optometry Faculty of Biology, Medicine and Health University of Manchester Manchester UK; ^4^ Division of Molecular and Cellular Function Faculty of Biology, Medicine and Health University of Manchester Manchester UK; ^5^ Navarrabiomed Complejo Hospitalario de Navarra (CHN) Instituto de Investigación Sanitaria de Navarra (IdiSNA) Universidad Pública de Navarra (UPNA) Pamplona Spain

**Keywords:** fibronectin, melanoma, MITF, phenotype cooperativity, phenotype plasticity

## Abstract

A major challenge for managing melanoma is its tumour heterogeneity based on individual co‐existing melanoma cell phenotypes. These phenotypes display variable responses to standard therapies, and they drive individual steps of melanoma progression; hence, understanding their behaviour is imperative. Melanoma phenotypes are defined by distinct transcriptional states, which relate to different melanocyte lineage development phases, ranging from a mesenchymal, neural crest‐like to a proliferative, melanocytic phenotype. It is thought that adaptive phenotype plasticity based on transcriptional reprogramming drives melanoma progression, but at which stage individual phenotypes dominate and moreover, how they interact is poorly understood. We monitored melanocytic and mesenchymal phenotypes throughout melanoma progression and detected transcriptional reprogramming at different stages, with a gain in mesenchymal traits in circulating melanoma cells (CTCs) and proliferative features in metastatic tumours. Intriguingly, we found that distinct phenotype populations interact in a cooperative manner, which generates tumours of greater “fitness,” supports CTCs and expands organotropic cues in metastases. Fibronectin, expressed in mesenchymal cells, acts as key player in cooperativity and promotes survival of melanocytic cells. Our data reveal an important role for inter‐phenotype communications at various stages of disease progression, suggesting these communications could act as therapeutic target.

**Significance**

Different melanoma cell phenotypes co‐exist within heterogeneous tumours, and apart from affecting responses to therapies, they drive distinct steps of melanoma progression; hence, understanding phenotype behaviour is imperative for better prognostic assessments. While phenotype plasticity based on transcriptional reprogramming has been studied extensively, little is known about phenotype interactions in heterogeneous tumours. We demonstrate here that inter‐phenotype communications contribute to phenotype plasticity, and result in cooperative behaviour, which accelerates tumour growth and supports melanoma cell dissemination and metastasis. We reveal an important role for inter‐phenotype communications at various stages of disease progression, suggesting these communications could act as therapeutic target.

## 1 INTRODUCTION

Intra‐tumour heterogeneity, reflected in the co‐existence of different cancer cell phenotypes within a tumour, is an inherent property of cancer. It is thought that transcriptional reprogramming establishes these phenotypes and dynamic reprogramming occurs throughout tumour progression. Epithelial–mesenchymal transition (EMT), which generates invasive epithelial‐derived phenotypes, is the most established concept of these dynamics.

In melanoma, which is not of epithelial origin, plasticity between cells of so‐called “proliferative” and “invasive” phenotypes had therefore been proposed as equivalent to EMT (Hoek et al., 2008). These two phenotype subpopulations are not defined by particular genetic lesions, but can be identified in melanoma tumours by their specific “proliferative” and “invasive” gene expression signatures (Rambow et al., 2018; Widmer et al., 2012). However, single cell analysis has identified melanoma cells simultaneously expressing genes corresponding to the “proliferative” or “invasive” signatures (Ennen et al., 2017; Tirosh et al., 2016), suggesting the existence of additional “transition” states. Indeed, recent studies revealed that the so‐called “proliferative” and “invasive” states are rather part of a series of melanoma cell subpopulations that transcriptionally relate to distinct melanocyte lineage development states (Rambow et al., 2018; Tsoi et al., 2018). Hence, we proposed a melanoma “phenotype plasticity model,” in which transcriptional reprogramming can occur along a differentiation/de‐differentiation gradient ranging from a melanocytic to a mesenchymal, neural crest‐like phenotype (Arozarena & Wellbrock, 2019). The respective transcriptional states are identifiable by classifiers such as MITF for the melanocytic phenotype, and SMADs, NFκB, AP1 and TEADs as upstream regulators of the mesenchymal phenotype transcriptome.

MITF is a melanocyte lineage‐specific transcription factor that rules the expression of pigmentation, but also cell cycle regulator genes; hence, so‐called MITF^high^ melanocytic phenotype cells are considered to be proliferative (Wellbrock & Arozarena, 2015). Low MITF expression correlates with de‐differentiation, and the MITF^low^ mesenchymal phenotype is stated to be invasive, because cells of this phenotype tend to show increased ability to invade matrigel in response to FCS (Carreira et al., 2006; Verfaillie et al., 2015; Widmer et al., 2012). This behaviour is probably due to the distinct expression of FCS‐responsive receptors induced by SMADs, TEADs or AP1 in the mesenchymal phenotype (Arozarena & Wellbrock, 2019; Miskolczi et al., 2018; Rambow et al., 2015). Thus, discrete biological properties have been assigned to the different melanoma cell phenotypes, and this categorization has been extrapolated to aid the definition of tumour phenotypes. However, intra‐tumour heterogeneity leads to the co‐existence of these phenotypes (Tirosh et al., 2016; Verfaillie et al., 2015), and phenotype heterogeneity can lead to cooperative behaviour during tumour development (Tabassum & Polyak, 2015). We showed recently that cooperative interactions between melanoma phenotypes impact on melanoma cell invasion (Chapman et al., 2014), but how communications between these phenotypes contribute to melanoma growth and progression is unknown.

To fully dissect the contribution of phenotype plasticity and cooperativity in melanoma, we analysed the impact of heterogeneity on melanocytic and mesenchymal phenotypes during individual steps of melanoma development and progression.

## 2 METHODS

For more detailed information, see Supporting Information Materials and Methods.

### 2.1 Cell lines

WM266‐4 cells were bought from the American Type Culture Collection, and 501mel cells were a gift from Steve Rosenberg (NCI, MD); all were obtained in 2008. WM266‐4‐GFP, 501mel‐mCherry and WM266‐4‐GFP FN‐kD have been previously described (Chapman et al., 2014) and had been generated with the Block‐iT Pol II miR RNAi expression vector kit (Invitrogen). All cell lines were authenticated in house by short tandem repeat profiling in 2018. These cell lines were grown in DMEM/10% FCS (PAA) as described previously (Wellbrock & Schartl, 2000).

### 2.2 Animal work

Animal procedures involving animals were ethically approved and carried out under licence in accordance with the UK Home Office Animals (Scientific Procedures) Act (1986) and guidelines of the Committee of the National Cancer Research Institute for the Welfare and Use of Animals in Cancer Research (Workman et al., 2010). Subcutaneous xenografts were established in NSG mice by injecting cells in serum‐free DMEM subcutaneously. Mice were weighed and tumours measured until they reached 1,000 mm^3^. Tumours, liver and lungs were harvested and fixed in formalin for immunohistochemistry. For the imaging studies, the indicated number of cells in PBS was implanted i.v.

### 2.3 Statistical analysis

If not indicated otherwise, data represent the results for assays performed in triplicate, with error bars to represent errors from the mean. Statistics were performed using GraphPad Prism version 8.00 for Mac OS, GraphPad Software, San Diego, California USA. If not indicated otherwise, one‐way ANOVA with Tukeys's post hoc test was used for bar graph analyses and the mixed effects model to analyse tumour growth over time. Where indicated, values are mean ± *SEM*. *p < .05; **p < .01; ***p < .001.

## 3 RESULTS

### 3.1 High FN1 expression in the mesenchymal phenotype accelerates the onset of melanoma growth

To assess the behaviour of different phenotypes, we chose melanocytic 501mel cells and mesenchymal WM266‐4 cells. Both cell lines have been widely used to study melanoma plasticity (Carreira et al., 2006; Cheli et al., 2011; Miskolczi et al., 2018; Ohanna et al., 2011; Strub et al., 2011), and because the respective transcriptional states linked to MITF in melanocytic 501mel cells or related to SMADs and AP1 in mesenchymal WM266‐4 cells are maintained when in culture (Figure S1a), they represent reliable models for each individual transcriptional phenotype.

**FIGURE 1:**
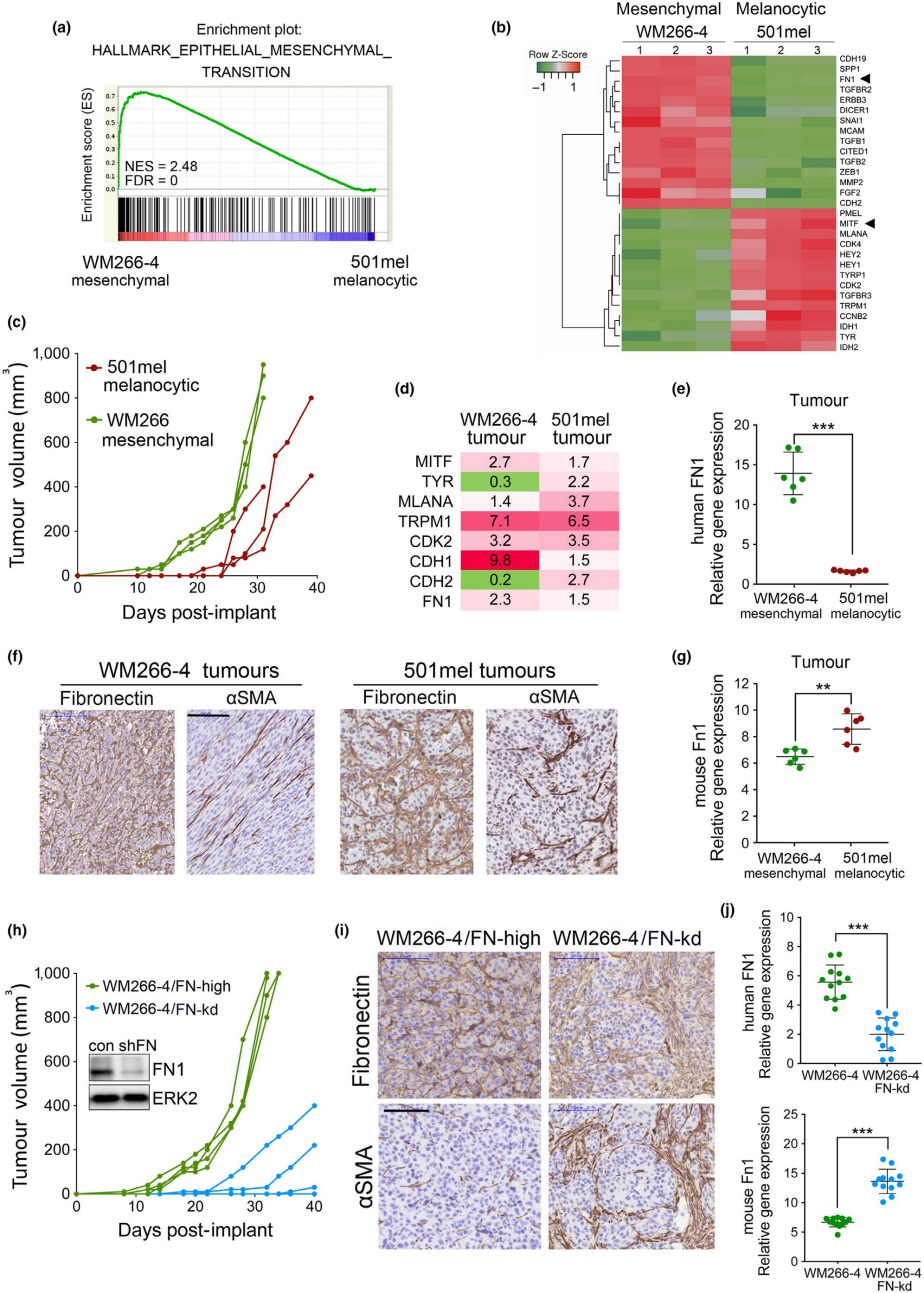
FN1 accelerates the onset of melanoma growth. (a) GSEA plot of EMT Hallmark (Liberzon et al., 2015) for 501mel‐mCherry and WM266‐4‐GFP cells. (b) Expression heatmap showing gene subset with the highest up‐ and down‐regulation in 501mel‐mCherry versus WM266‐4‐GFP cells. (c) Tumour volumes over 31 days in mice (*n* = 3 mice/group) injected with 501mel‐mCherry and WM266‐4‐GFP cells. (d) Fold change in mRNA expression in indicated tumours when compared to cell lines. (e) qRT‐PCR analysis of human FN1 expression in indicated tumours. (f) IHC for fibronectin and αSMA in indicated tumours. Scale bar: 100 µm. (g) qRT‐PCR analysis of mouse Fn1 expression as in (e). (h) Tumour volumes over a period of 40 days in mice (*n* = 4 mice/group) injected with WM266‐4‐GFP or WM266‐4 FN‐kd‐GFP cells. Expression of FN1 in both cell lines was analysed by Western blotting. ERK2 served as loading control. (i) IHC for fibronectin and αSMA in WM266‐4‐GFP tumours (day 32) and WM266‐4 FN‐kd‐GFP tumours (day 40). Scale bar: 100 µm. (j) qRT‐PCR analysis of human FN1 and mouse Fn1 expression in the respective tumours. **p < .01; ***p < .001, for GSEA: FDR < 0.0001

In line with their melanocytic phenotype, 501mel cells are identified by the Hoek (Widmer et al., 2012) as well as the Verfallie (Verfaillie et al., 2015) “proliferative” signature, whereas mesenchymal WM266‐4 cells are significantly enriched in “invasive” signature genes (Figure S1b–d). Expression of GFP or mCherry did not change the respective phenotypes with regard to their functional behaviour, or melanocytic or mesenchymal features (Figure 1a,b and Figure S1c,d). Thus, these cell lines demonstrate all characteristics of the respective melanocytic/proliferative or mesenchymal/invasive phenotypes of which MITF expression levels serve as classifier (see Figure 1b).

In line with previous observations (Caramel et al., 2013; Cheli et al., 2011; Rambow et al., 2015; Wardwell‐Ozgo et al., 2014), the onset of growth of melanocytic 501mel tumours was delayed compared to mesenchymal WM266‐4 tumours (Figure 1c and Figure S2a). Expression of MITF and its target genes linked to differentiation and proliferation was up‐regulated in all growing tumours compared to the initially injected cells (Figure 1d and Figure S2b), which is in line with the requirement of MITF for tumour growth (Simmons, Pierce, Al‐Ejeh, & Boyle, 2017). Intriguingly, the expression of human fibronectin (FN1) was also increased (Figure 1d), suggesting that it is also favourable for tumour growth.

Immunohistochemistry (IHC) analysis using a fibronectin antibody with reactivity to mouse and human revealed a similar staining intensity in 501mel and WM266‐4 tumours despite a significant lower mRNA expression of human FN1 in 501mel tumours (Figure 1e,f). However, murine Fn1 expression was elevated in 501mel tumours (Figure 1g), and this correlated with an abundance of cells positive for the cancer‐associated fibroblast (CAF) marker αSMA (Figure 1f). We found that mouse Fn1 could serve as adhesion substrate and activate adhesion signalling in 501mel cells (Figure S2c,d), supporting the idea that fibronectin derived from murine CAFs might aid tumour growth. This idea was further corroborated by findings with WM266‐4 cells in which FN1 was depleted by RNAi; the loss of FN1 profoundly delayed tumour growth (Figure 1h and Figure S2e). However, tumours that eventually started growing contained abundant αSMA‐positive CAFs correlating with increased host Fn1 expression (Figure 1i,j). At the cellular level, fibronectin supported melanoma cell aggregation (Figure S2f,g) and protected WM266‐4 cells from apoptosis, which was also seen for low FN1 expressing 501mel cells (Figure S2h,i).

### 3.2 Mesenchymal, but also melanocytic phenotype cells are sufficient to seed metastases

**FIGURE 2:**
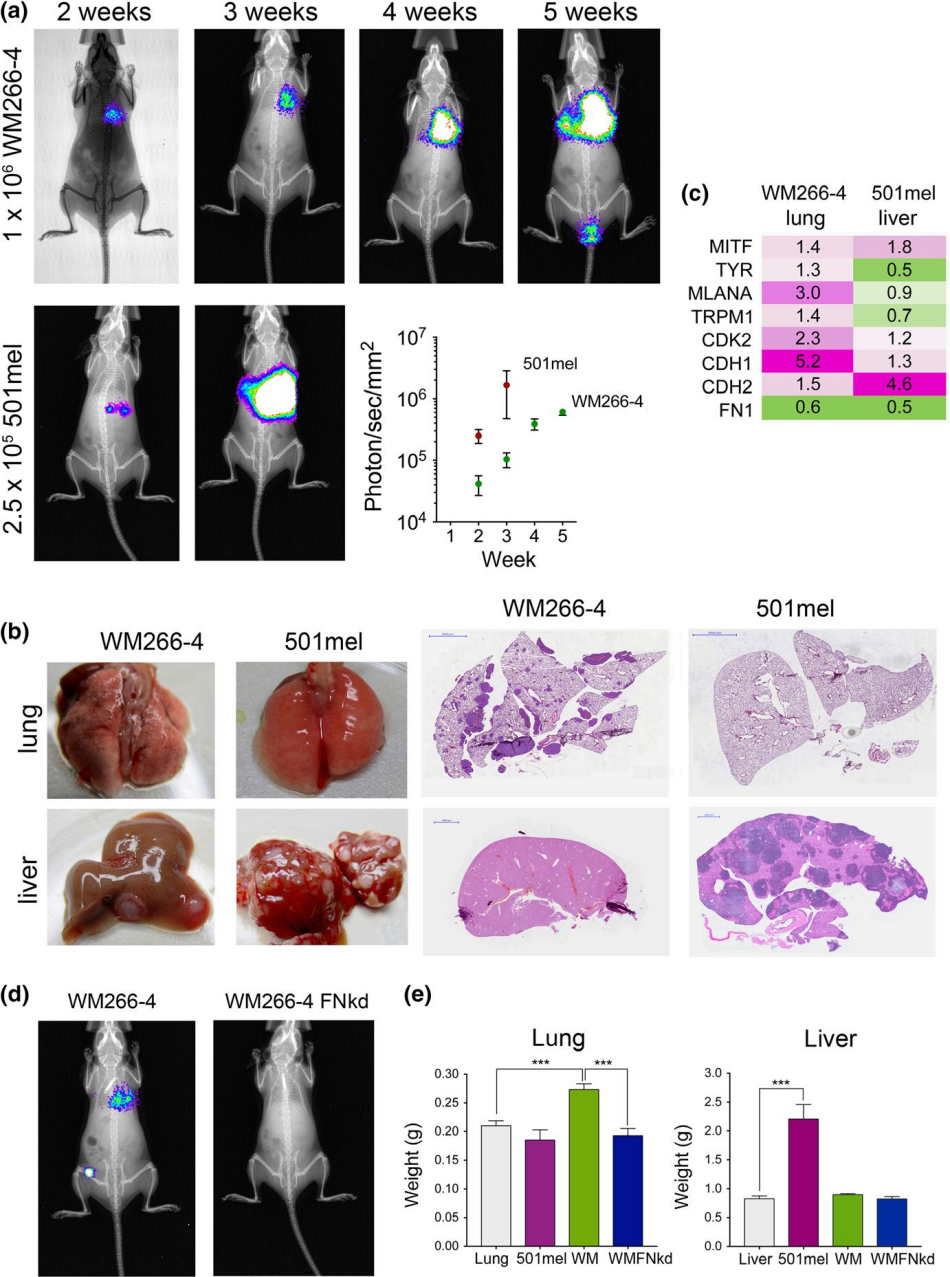
Mesenchymal and melanocytic melanoma cells seed metastases. (a) Representative images of in vivo bioluminescence imaging in NSG mice injected i.v. with luciferase expressing WM266‐4‐GFP or 501mel‐mCherry cells. Quantitation of imaging signals of serial images is shown (*n* = 4 mice/group). (b) Photographs and H&E staining of livers and lungs isolated from mice described in (a). Scale bar: 2000 µm. (c) qRT‐PCR analysis of indicated genes and in indicated cell lines and corresponding metastases. The mean fold change (*n* = 3) is indicated, cut‐off *p* < .05. (d) Representative images of in vivo bioluminescence imaging of metastases in NSG mice injected IV with luciferase expressing WM266‐4‐GFP or WM266‐4 FN‐kd‐GFP cells (*n* = 3 mice/group). (e) Lung and liver weights from mice injected with the indicated cell lines (*n* = 3 mice/group). Normal lungs and livers served as control. One‐way ANOVA, ***p < .001

We confirmed (Eccles et al., 2008; Mills et al., 2002) that mesenchymal WM266‐4 cells efficiently seed the lung after tail vein injection, however melanocytic 501mel cells seeded the liver instead (Figure 2a‐b). While due to this organotropism previous studies might have missed the metastatic potential of 501mel cells, it should be mentioned that compared to others (Ohanna et al., 2011; Tichet et al., 2015), we used NSG mice for these experiments, which suggests a possible contribution of the immune environment in defining the metastatic potential of 501mel cells.

Expression of MITF and its target gene CDK2 was increased in both, lung and liver metastases (Figure 2c), which agrees with the fact that MITF is required for metastasis outgrowth (Simmons et al., 2017). On the other hand, FN1 expression was down‐regulated (Figure 2c), and with 501mel cells expressing hardly detectable basal levels of FN1 (Figure S2g), this strongly suggested that cell‐derived FN1 is not a relevant factor for metastatic seeding of the liver by 501mel cells. On the other hand, using WM266‐4 FN‐kd cells revealed that WM266‐4‐derived FN1 is required for their ability to establish lung metastasis (Figure 2d). Lung and liver weights (Figure 2e) reflected that both phenotypes were able to produce experimental metastasis and that WM266‐4 cells required FN1 to seed the lung.

### 3.3 Melanocytic phenotype cells cooperate with mesenchymal phenotype cells in tumour growth in a FN‐dependent manner

**FIGURE 3:**
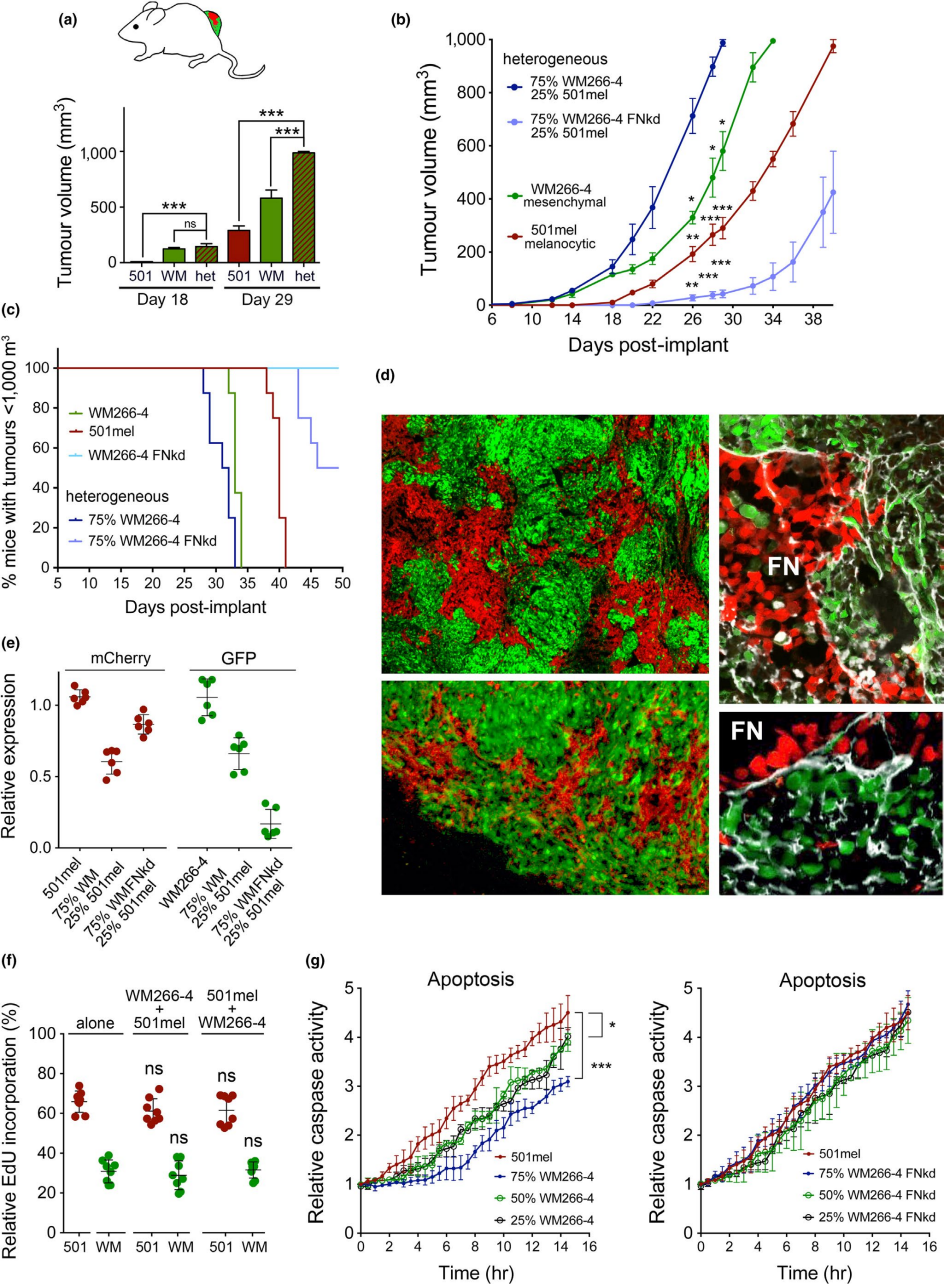
Cooperativity between phenotypes occurs in a FN‐dependent manner. (a) Tumour volumes at days 18 and 29 in mice (*n* = 4 mice/group) injected with 501mel‐mCherry or WM266‐4‐GFP cells. (b) Tumour volumes over a period of 40 days in mice (*n* = 4 mice/group) described in (a). Statistics are comparing heterogeneous 501mel‐mCherry/WM266‐4‐GFP tumours with the indicated tumours. (c) Tumour‐free survival. (d) Direct fluorescence imaging of frozen tumour sections derived from mice injected with 501mel‐mCherry or WM266‐4‐GFP cells. Sections were stained for fibronectin expression using a pan‐ fibronectin antibody. (e) qRT‐PCR analysis of GFP and mCherry expression in tumours from mice injected with 501mel‐mCherry or WM266‐4‐GFP cells either alone or in combination as indicated. (f) EdU incorporation into 501mel‐mCherry or WM266‐4‐GFP cells when either co‐cultured with themselves or with each other (either 501mel or WM266‐4 cells in insert). (g) Relative NucView^®^ 488‐caspase activity over time was measured in aggregated 501mel‐mCherry cells in the presence of increasing amounts WM266‐4‐GFP or WM266‐4 FN‐kd‐GFP cells using the Incucyte imaging system. *p < .05; ***p < .001; ***p < .001

To assess how phenotype heterogeneity affects melanoma growth, we analysed xenografts grown by either WM266‐4 or 501mel cells or a heterogeneous mixture of both cell lines; due to the high proliferative activity of 501mel cells, we chose a ratio of 25%–75% WM266‐4 cells. Tumour growth onset of heterogeneous tumours was not affected when compared to WM266‐4 tumours (Figure 3a), but heterogeneous tumours grew significantly faster (Figure 3b and Figure S3) and had reached a greater volume at 29 days than WM266‐4 tumours (Figure 3a). Heterogeneous tumours containing WM266‐4 FN‐kd showed delayed tumour growth onset (Figure 3b), indicating that FN1 is also important for growth in heterogeneous tumours, which was also reflected in tumour‐free survival (Figure 3c).

Heterogeneous tumours were composed of red 501mel and green WM266‐4 subpopulations growing in clusters, but occasionally, particularly at tumour edges, the subpopulations intermixed (Figure 3d). Fibronectin co‐localized with WM266‐4‐GFP cells was enriched between the populations and also detectable in areas where 501mel‐mCherry cells grew (Figure 3d). A more detailed analysis of tumour composition analysing GFP‐ or mCherry‐mRNA expression revealed that although initially injected at 25%, 501mel cells made up 50% of heterogeneous tumours when they reached ~1000 mm^3^ (Figure 3e). Even more striking, in tumours that contained FN1‐deficient WM266‐4 cells, 501mel cells outgrew the incompatible WM266‐4 FN‐kd cell population (Figure 3e). At the cellular level, there was no proliferative advantage when 501mel cells were co‐cultured with WM266‐4 cells (Figure 3f), but there was a significant effect on survival in heterogeneous melanoma spheres and this effect was dependent on the ability of WM266‐4 cells to express FN1 (Figure 3g).

### 3.4 Cooperativity in heterogeneous tumours results in “phenotype adaptation” compatible with enhanced tumour growth

Bulk RNA sequencing (RNA‐seq) has revealed that “invasive,” “neural crest stem cell‐like” and “pigmented” phenotypes co‐exist within a tumour and can be identified by their respective expression signatures (Rambow et al., 2018). Following this approach, we assessed the impact of cooperativity on individual phenotype populations during tumour growth.

**FIGURE 4:**
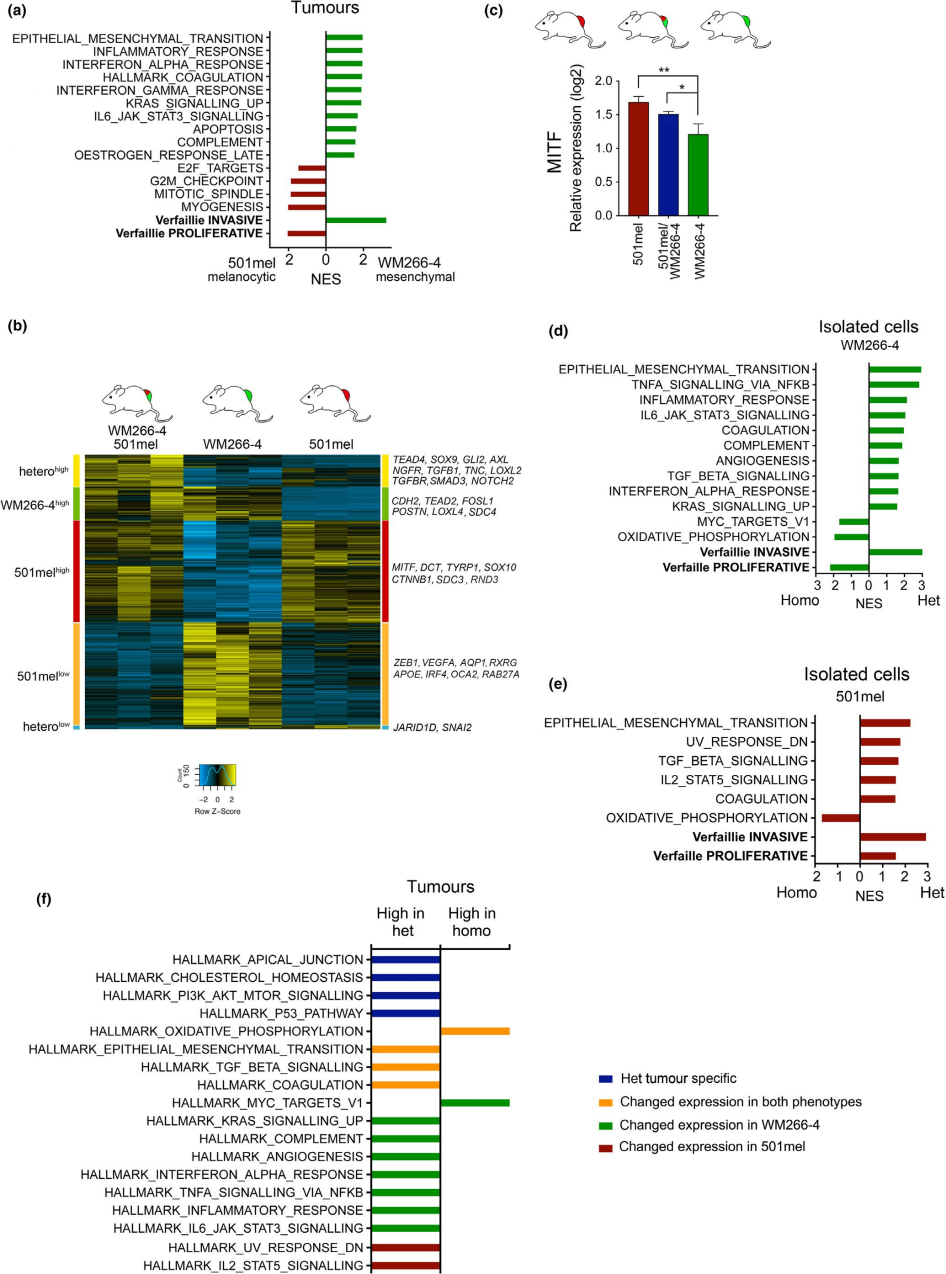
Cooperativity in heterogeneous tumours results in “phenotype adaptation”. (a) Functional characteristics of 501mel‐mCherry or WM266‐4‐GFP tumours revealed by GSEA using the Verfaillie “invasive” and “proliferative” signature and the MSigDB hallmark gene set collection (Liberzon et al., 2015). (b) Hierarchical clustering heatmap of 6,176 genes that are differentially expressed between 501mel‐mCherry, WM266‐4‐GFP and heterogeneous tumours. (c) qRT‐PCR analysis of MITF expression in the indicated tumours. (d) Functional characteristics of WM266‐4‐GFP cells sorted either from homogeneous WM266‐4 tumours or from heterogeneous tumours revealed by GSEA. (e) Functional characteristics of 501mel‐mCherry cells sorted either from homogeneous 501mel tumours or from heterogeneous tumours revealed by GSEA. (f) Functional characteristics of heterogeneous tumours. Unique characteristics of heterogeneous tumours are in blue. Transcriptional changes induced in both subpopulations are in orange. Hallmarks of heterogeneous tumours derived from changes in expression in WM266‐4 cells (green), and 510mel cells (red) are also indicated. **p < .01; ***p < .001. For GSEA analysis: FDR < 0.05

In “homogenous” WM266‐4 and 501mel tumours, the phenotype corresponding to each cell line was retained (Figure 4a and see Figure S1b). Unsupervised clustering of RNA‐seq data from “homogeneous” and “heterogeneous” tumours distinguished the different tumour groups and identified 3 clusters, which in heterogeneous tumours were linked to expression patterns of 501mel (501mel^high^, 501mel^low^) and WM266‐4 (WM266‐4^high^) tumours, respectively (Figure 4b). However, “heterogeneous” tumours also expressed two clusters with uniquely down‐regulated (hetero^low^) and up‐regulated (hetero^high^) genes, respectively. The group of down‐regulated genes comprised only 69 genes amongst them were *JARID1D* (*KDM5D*) and *SNAI2*. The cluster of uniquely up‐regulated genes was enriched in established “invasive‐phenotype” markers like *AXL, SOX9, TGFB* and *NGFR* (Figure 4b). Indeed, heterogeneous tumours were enriched for EMT and Verfaillie “invasive” signatures even relative to WM266‐4 tumours (Figure S4a,b), but MITF expression was higher than in WM266‐4 tumours (Figure 4c).

To assess specific changes in the mesenchymal WM266‐4 populations, we FACS‐sorted these from homogeneous or heterogeneous tumours and found that MITF expression was similar under both conditions (Figure S4c,d). Nevertheless, EMT, TNFα, TGFβ and inflammatory response signatures were enriched in WM266‐4 cells in a heterogeneous setting (Figure 4d). Thus, the presence of melanocytic phenotype 501mel cells had stimulated a further up‐regulation of mesenchymal features in WM266‐4 cells, which is also reflected in enrichment for the Verfaillie “invasive” and reduction of the “proliferative” signature (Figure 4d).

In isolated 501mel cells, the Verfaillie “proliferative” signature was up‐regulated, but intriguingly the subpopulation had also acquired hallmarks of EMT and TGFβ signalling and was enriched for the Verfaillie “invasive” signature (Figure 4e). Thus, while maintaining characteristics of the melanocytic phenotype, 501mel cells had also gained mesenchymal features in the presence of WM266‐4 cells. This gain in mesenchymal features was correlated with an up‐regulation of AP1 complex factors such as JUN, FOS and FOSL1 as well as TEAD1, whereas MITF expression was unchanged (Figure S4e).

In heterogeneous tumours, both phenotype subpopulations had down‐regulated oxidative phosphorylation (Figure 4d,e, and Figure S4a,b), suggesting a change in metabolic activity compatible with enhanced tumour growth. Individual contributions of the two phenotypes in “heterogeneous” tumours resulted in major changes in cell–cell junctions and ECM interactions with increased expression of *FN1*, *TNC* and matrix re‐modellers, and alterations in the repertoire of collagens and adhesion receptors (Figure 4f and Tables S1–S3).

Overall, transcriptional plasticity in both phenotype subpopulations within heterogeneous tumours resulted in up‐regulation of functional programmes that established tumours of greater “fitness” with enhanced growth and reduced cell death linked to increased ECM dynamics and cell adhesion. Because we have analysed a pool and not single cells, we are not able to distinguish between general transcriptional changes in all cells of one subpopulation or specific changes in just some cells. The latter however appears more likely, because close interaction between the different phenotypes does not occur throughout the whole tumour (see Figure 3d).

### 3.5 Fibronectin‐mediated cooperativity enhances CTC persistence

**FIGURE 5:**
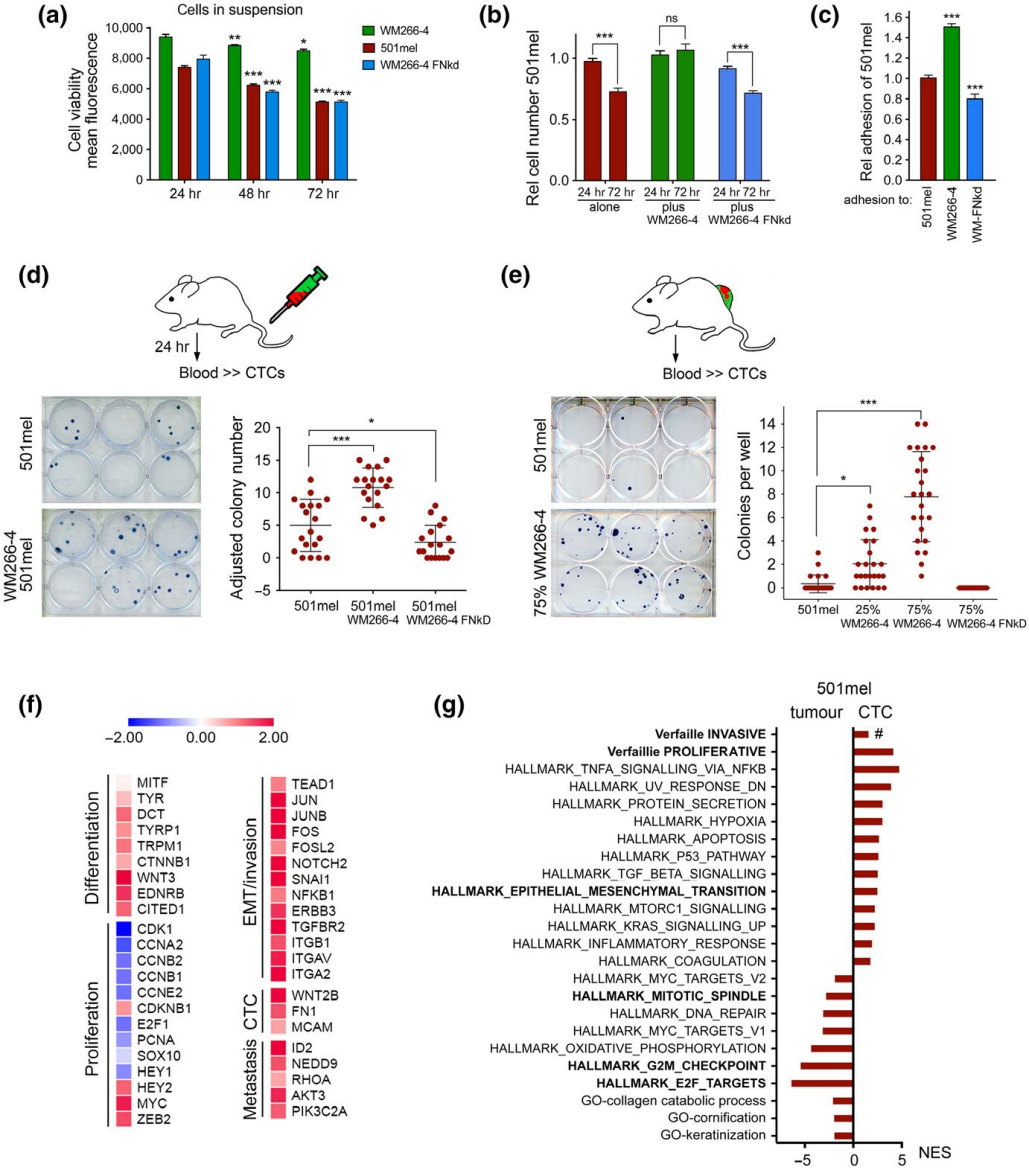
Fibronectin‐mediated cooperativity enhances CTC persistence. (a) Quantification of cell viability of 501mel‐mCherry, WM266‐4‐GFP or WM266‐4 FN‐kd‐GFP cells in suspension. (b) Relative cell number of 501mel‐mCherry cells under anoikis conditions (plates coated with 3% agarose) either alone or co‐cultured with WM266‐4‐GFP or WM266‐4 FN‐kd‐GFP cells. (c) Relative adhesion of mCherry or GFP expressing 501mel to indicated cells. (d) Quantification of 501mel CTCs isolated from mice (*n* = 3 mice/group) 24 h after IV injection with the indicated cell lines (either 100% or 50% 501mel‐mCherry cells in heterogeneous injections). The number of colonies formed from mice injected with 50% cells has been adjusted to 100%. (e) Quantification of 501mel CTCs isolated from mice (*n* = 4 mice/group). Blood was isolated from mice with 501mel‐mCherry tumours at day 38, for heterogeneous tumours at day 28 and for WM266‐4 FN‐kd‐GFP tumours at day 50. (f) Heatmap of fold induction of gene expression in CTCs compared to tumours. (g) Functional characteristics of 501mel‐mCherry CTCs and 501mel‐mCherry tumours revealed by GSEA using the MSigDB hallmark and Gene Ontology (GO) term gene set collection, as well as the Verfaillie proliferative and invasive signatures

Because we found that WM266‐4 cells can provide a survival advantage for 501mel cells partly linked to adhesion signalling (Figure 3g), we argued that this might also be relevant for circulating melanoma cells (CTCs). We confirmed that WM266‐4 cells were more resistant to anoikis and provided a survival advantage to 501mel cells, which was partly dependent on FN1 expression (Figure 5a,b). Furthermore, 501mel cells adhered stronger to WM266‐4 cells than to themselves, and this was abolished in the absence of FN1 (Figure 5c).

We next injected 501mel cells i.v. either alone or with WM266‐4 cells and after 24h analysed blood for the presence of melanoma cells by colony formation. In this experimental approach, only 501mel cells grew as colonies, as WM266‐4 cells were highly inefficient to initiate a colony from a single cell (data not shown). We found that the presence of WM266‐4 cells significantly increased the number of 501mel colonies, but WM266‐4 FN‐kd cells were unable to do so (Figure 5d).

In tumour‐bearing mice, we found a dose‐dependent increase in colonies formed from circulating 501mel cells under heterogeneous conditions (Figure 5e). This could be due to the fact that in heterogeneous tumours, 501mel cells had acquired features of EMT (see Figure 4e). For tumours grown with 501mel and WM266‐4 FN‐kd cells, CTCs were below the detection level which might be due to a lower number of 501mel cells present in these tumours or that WM266‐4 FN‐kd cells had an inhibitory effect on 501mel cell dissemination.

We were intrigued to detect CTCs from homogeneous 501mel tumours, because in vitro 501mel cells are weakly invasive (Arozarena et al., 2011; Carreira et al., 2006). Reasons for this altered behaviour in vivo could be that 501mel cell invasion is supported by the host microenvironment, and indeed, we found that mouse fibronectin has the potential to enhance 501mel cell invasion in vitro (Figure S5). In addition, we argued that phenotype plasticity in homogenous 501mel tumours could enable 501mel cells to access the circulation. To address this, we isolated 501mel CTCs from tumour‐bearing mice and performed RNA‐seq. The isolated CTCs displayed up‐regulation of *FN1* (and its integrin receptors), as well as *WNT2* and *MCAM* (Figure 5f), all previously implicated in CTCs (Rapanotti et al., 2017; Yu et al., 2012).

Overall, the CTCs derived from melanocytic 501mel cells displayed features of a mesenchymal phenotype in which TGFB, TNFA/NFKB and the inflammatory response signatures were enriched (Figure 5g). Intriguingly however, enrichment of the Verfaillie “invasive” signature did not reach significance (FDR q = 0.055489495) (Figure 5g), and 501mel CTCs remained negative for *AXL* and *WNT5A*, the most established markers of the “invasive” signature. On the other hand, down‐regulation of a large group of keratins characteristic for EMT was observed, and CTCs had up‐regulated an EMT signature (Figure 5g and Table S4). This suggests that melanoma cells can feature an EMT/mesenchymal phenotype without having to adopt all characteristics of the “invasive” signature. Indeed, while the expression of EMT upstream regulators like TEAD1 and AP1 factors FOS, JUNB and JUN was up‐regulated in CTCs, MITF expression was unchanged (Figure 5f).

501mel CTCs also displayed down‐regulation of cell cycle progression genes such as *CDK1*, *CCNA* and *CCNB*, and signatures related to E2F targets, G2M checkpoint and mitotic spindle were down‐regulated (Figure 5f,g). This suggested reduced proliferative activity in 501mel CTCs, yet they showed a significant enrichment for the Verfaillie “proliferative” signature. An explanation for this could be that genes linked to melanocyte differentiation (see Figure 5f) and possibly other cell cycle‐independent genes contained within the “proliferative” signature were actually up‐regulated in CTCs.

### 3.6 Phenotype cooperativity expands organotropic cues

**FIGURE 6:**
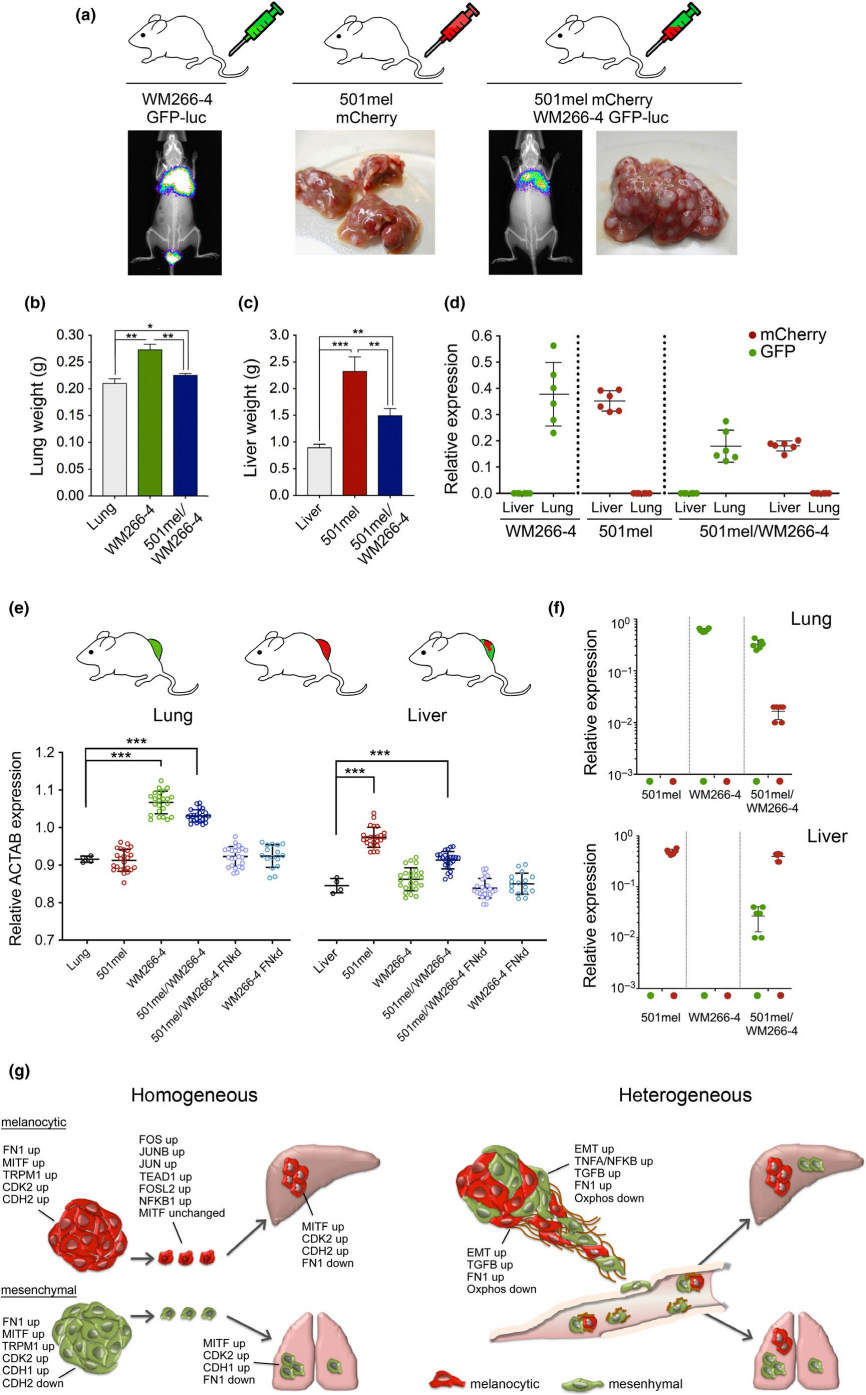
Phenotype cooperativity expands organotropic cues. (a) NSG mice (3 mice/group) were injected IV with either luciferase expressing WM266‐4‐GFP or 501mel‐mCherry cells or a 50/50% mix. Mice were imaged for in vivo bioluminescence at week 5 after injection, and livers were extracted. Representative images are shown. (b) Lung, (c) liver weights from mice injected as in (a). Normal lungs and livers served as control. (d) qRT‐PCR analysis of GFP and mCherry expression in lungs and livers from mice defined in (a). (e) qRT‐PCR analysis for human ACTAB expression in lungs and livers from tumour‐bearing mice (*n* = 3 mice/group) induced by 501mel‐mCherry or WM266‐4‐GFP or WM266‐4 FN‐Kd‐GFP cells either alone or in combination. (f) qRT‐PCR analysis of GFP and mCherry expression in lungs or livers from mice described in (e). (g) Model depicting transcriptional changes and cooperative effects in homogeneous melanocytic 501mel‐mCherry or mesenchymal WM266‐4‐GFP and heterogeneous tumours during melanoma progression. *p < .05; **p < .001; ***p < .001

Finally, we wanted to assess whether cooperativity also occurs during the establishment and growth of metastases. For this, we injected mice with luciferase expressing WM266‐4‐GFP cells and luciferase‐negative 501mel‐mCherry cells. We could detect lung metastasis when WM266‐4 cells were injected alone and co‐injected with 501mel cells (Figure 6a). Likewise, we could detect liver metastases in mice injected with 501mel cells in both settings (Figure 6a). Because in the co‐injection only 50% of each subpopulation was present, the luciferase signal from WM266‐4 cells in the lung was significantly stronger in the homogeneous than in the heterogeneous setting, and this was also reflected in the lung weight (Figure 6a,b). Similarly, the liver weight of co‐injected mice lay in between that of control and singly injected mice (Figure 6c). Analysis of lungs and livers for the presence of GFP or mCherry signals revealed that no cooperativity had occurred during the immediate steps of metastasis. Instead, the two subpopulations had followed their organotropic cues in order to seed metastasis (Figure 6d).

In the tail vein experiment, heterogeneity only occurs for a limited time after cells have been injected, but when both subpopulations reach an organ and one population encounters an environment that is inappropriate for it to seed and grow, then a selection occurs and heterogeneity is lost for the remaining time of the experiment. We therefore wondered, whether in mice that had grown heterogeneous tumours over a longer period of time cooperativity might be visible, because under these conditions, heterogeneous subpopulations of cells should be disseminated constantly throughout the experiment.

We first analysed mice that had developed homogeneous 501mel or WM266‐4 tumours as well as mice with heterogeneous tumours for the presence of human ACTAB expression, and could confirm expression in livers of mice with 501mel and in lungs of mice with WM266‐4 tumours (Figure 6e). In mice bearing heterogeneous tumours, we detected ACTAB signals in the lung and in the liver (Figure 6e). We then analysed these organs for the presence of GFP and mCherry expressing cells, and found GFP signals in the lungs of mice with WM266‐4 as well as heterogeneous tumours, and mCherry signals in the livers of mice with 501mel or heterogeneous tumours (Figure 6f). Most strikingly though, in the mice that had developed heterogeneous tumours we could also detect signals for mCherry in the lung and GFP in the liver (Figure 6f), indicating that the constant release of heterogeneous circulating melanoma cells led to an expansion of organotropism and the cells could now successfully seed a previously incompatible tissue environment.

## 4 DISCUSSION

Increasing evidence indicates that tumours act as “communities,” whereby cooperative behaviour between individual phenotypes can contribute to tumour progression (Tabassum & Polyak, 2015). We showed previously that melanocytic MITF^high^ and mesenchymal MITF^low^ melanoma cells cooperate to drive invasion of melanoma in vivo (Chapman et al., 2014). For this, we chose a melanocytic MITF^high^ cell line and a mesenchymal MITF^low^ cell line, whereby MITF expression levels serve as classifier for these phenotypes. Comparison between these distinct cell lines is preferable to comparison of in vitro generated sub‐clones from an individual cell line in which MITF expression levels have been manipulated. This is because in vitro, in the absence of signalling from the tumour microenvironment, each phenotype‐specific epigenetic background is incompatible with sustained genetic manipulation of MITF expression. As such, ectopically over‐expressed MITF cannot induce differentiation and proliferation in mesenchymal MITF^low^ cells such as RPMI‐7951 or WM266‐4, and RNAi‐mediated MITF depletion in 501mel cells result in DNA damage and induction of senescence rather than establishing a mesenchymal state (Carreira et al., 2006; Cheli, Ohanna, Ballotti, & Bertolotto, 2010; Lauss et al., 2015; Strub et al., 2011; Vachtenheim, Novotna, & Ghanem, 2001; Wellbrock & Marais, 2005).

We show here that phenotype cooperativity can lead to earlier onset and faster growth of tumours. This cooperativity was partly dependent on cell‐derived FN1, which protects melanoma cells from anoikis (Boisvert‐Adamo & Aplin, 2006) and appears to provide a survival advantage for melanoma cells independent of their phenotype. We found that FN1 also impacted on the persistence of CTCs and is important for survival of cells in suspension, possibly by providing a “platform” for activating adhesion signalling (Boisvert‐Adamo & Aplin, 2006). Several studies have assessed the functional role of FN1 in melanoma cells in vitro and the role of niche and plasma fibronectin in the context of melanoma metastasis (von Au et al., 2013; Garmy‐Susini et al., 2010; Malik et al., 2010; Pasqualini, Bourdoulous, Koivunen, Woods, & Ruoslahti, 1996), but we reveal a new role for melanoma cell‐derived FN1, which due to cooperative effects accelerates the growth of heterogeneous tumours and enhances melanoma cell dissemination by acting on CTCs. In line with this, fibronectin is up‐regulated in pancreatic CTCs, where it is involved in anoikis protection (Yu et al., 2012). Furthermore, similar to melanoma (Khoja et al., 2014), CTC clusters are found in breast cancer, and their existence is dependent on the presence of plakoglobin, emphasizing the relevance of cell–cell adhesions (Aceto et al., 2014).

Cooperative behaviour can induce alterations in the metastatic potential of cancer cells. For instance, non‐metastatic breast cancer clones can cooperate to establish metastasis, which they are unable to do as individual clonal populations (Marusyk et al., 2014). Likewise, SCLC neuroendocrine and non‐neuroendocrine phenotypes are required for metastasis (Calbo et al., 2011). In our setting, both phenotypes were able to establish metastasis, but in a heterogeneous setting, their specific organotropic cues were expanded (Figure 6g). We do not know, whether the two phenotypes seeded individual secondary tumours or gave rise to heterogeneous metastases, but the idea of multiple distinct cell populations derived from a primary melanoma being able to act as founders of individual metastases is supported by findings from phylogenetic analyses (Sanborn et al., 2015).

The phenomenon of expanded organotropism could be due to changes in the metastatic niche and/or due to changes in the transcriptional state in heterogeneous CTCs. While we could not test the latter in heterogeneous CTC populations (the more complex sorting process resulted in poor RNA quality), we found high expression levels of SPARC, FN1 and TNC in 501mel cells from heterogeneous tumours when compared to cells from homogeneous tumours, and importantly, all three factors have been implicated in lung metastasis of melanoma (Fukunaga‐Kalabis et al., 2010; Tichet et al., 2015).

Possibly negative selection due to the lung‐specific immune environment contributes to the exclusion of 501mel cells. 501mel cells are widely used as the gold‐standard for non‐tumorigenic and non‐metastatic melanoma cells (Ohanna et al., 2011; Tichet et al., 2015). Indeed, they grow very ineffectively and are non‐metastatic in athymic nude mice, but we find that these properties are dramatically changed in an NSG background. Interestingly, factors secreted from senescent cells (Ohanna et al., 2011) or overexpression of SPARC (Tichet et al., 2015) enable 501mel cells to establish lung metastasis in nude mice, but whether these factors change the immune recognition of 501mel cells has not been assessed.

While there is increasing evidence for cooperativity at different stages, not much is known about the effect of phenotype communications on the dynamics of transcriptional states. We found that in heterogeneous tumours, the melanocytic and mesenchymal phenotypes adapt through phenotype plasticity with an overall trend of acquiring more mesenchymal features. In tumours, this resulted in melanocytic phenotype cells that still expressed high levels of MITF, but had up‐regulated EMT and invasive signatures and corresponding transcription factors such as TEAD1 and AP1 factors (summarized in Figure 6g). Our findings agree with gene expression analyses in melanoma that revealed a gradient of phenotypes (Tirosh et al., 2016) and cell states simultaneously expressing both signatures (Ennen et al., 2017). For epithelial cancers, a recent study in transgenic mice identified subpopulations that represent individual epithelial to mesenchymal transition states, which supports the idea that EMT occurs in a gradual manner (Pastushenko et al., 2018). We recently proposed a “phenotype plasticity model” in which a differentiated melanocyte and an un‐differentiated neural crest stem cell‐like state are epigenetically stable extremes (Arozarena & Wellbrock, 2019). In this model, complete switching from one to the other phenotype would require considerable “activation energy” and may therefore occur with very low frequency. Our data suggest that during melanoma growth and progression, phenotype adaptation occurs, whereby crucial transcriptional regulators of one phenotype are up‐regulated, while characteristics of the other phenotype are still maintained.

Understanding the dynamics and identifying drivers of the most relevant “transition” state(s) is crucial whether we want to improve our possibilities to predict tumour progression. Moreover, therapeutic intervention inevitably impacts on these dynamics and selects for certain cell states. We and others have shown that blocking drivers of relevant heterogeneous phenotypes can improve the outcome of targeted therapy in melanoma (Obenauf et al., 2015; Smith et al., 2017), and our data presented here suggest that blocking FN1‐mediated interactions can interfere with melanoma at various stages of disease progression.

## CONFLICT OF INTEREST

All authors declare no potential conflict of interest.

## Supporting information

Supplementary Material

Tables S1‐S4
