# Supplementary material for: Cooperative behaviour and phenotype plasticity evolve during melanoma progression
Source: Pigment Cell Melanoma Res. 2020 Mar 20;33(5):695–708. doi: 10.1111/pcmr.12873 (PMC7496243; doi:10.1111/pcmr.12873)
Supplement: Supplementary file 1 — Supplementary Material [file PCMR-33-695-s001.docx]

## Supplemental Information

**Cooperative behaviour and phenotype plasticity evolve during melanoma progression**

##

Emily J Rowling^1^, Zsofia Miskolczi^1^, Raghavendar Nagaraju^1^, Daniel J Wilcock^1^, Ping Wang^2^, Brian Telfer^3^, Yaoyong Li^4^, Irene Lasheras-Otero^5^, Marta Redondo-Muñoz^5^, Andrew D Sharrocks^4^, Imanol Arozarena^5^ and Claudia Wellbrock^1^

**
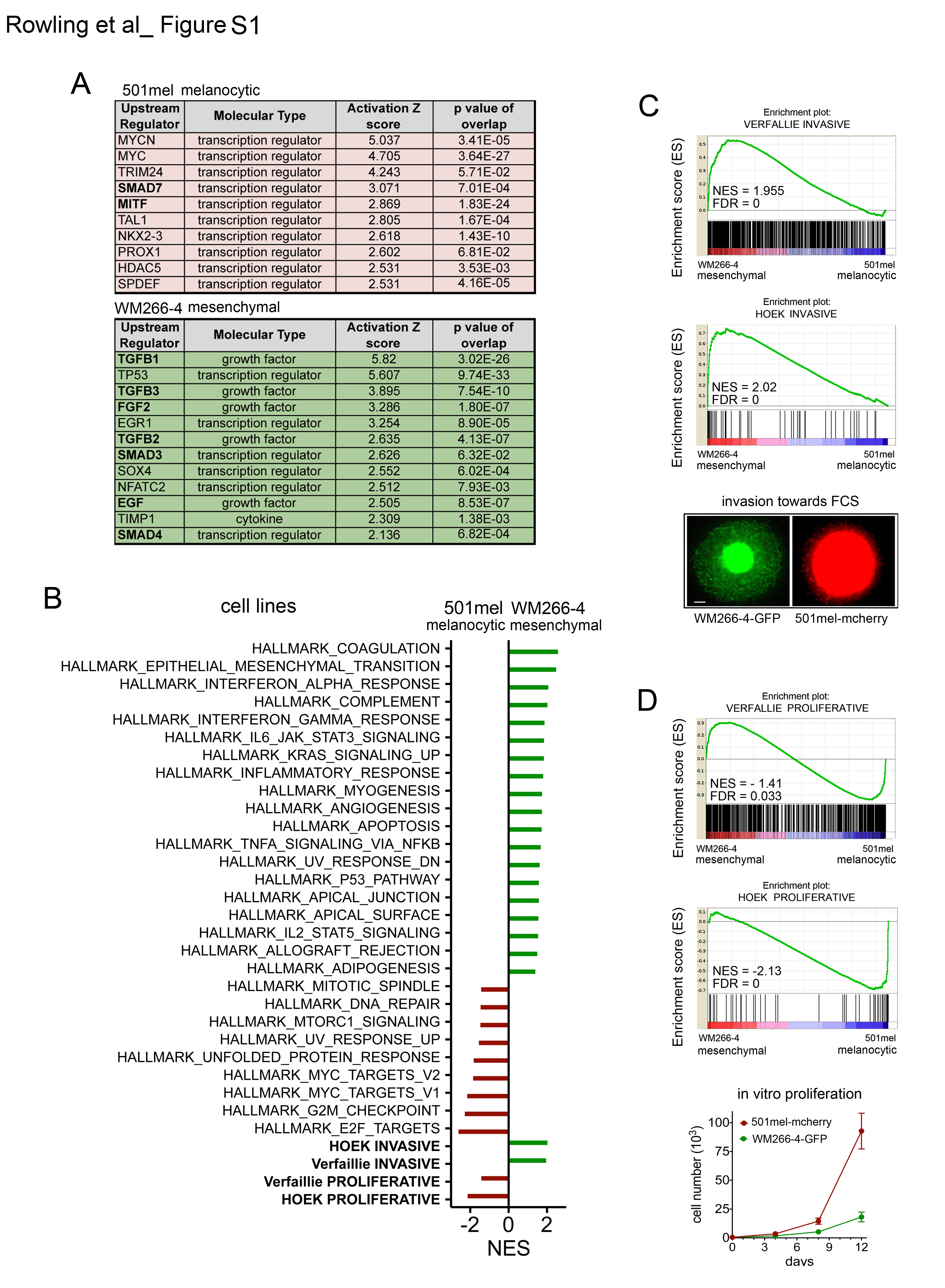
**

**Figure S1, rel. to Fig 1: Characterisation of melanocytic and mesenchymal cells**

(a) IPA Upstream Regulator Analysis of differentially expressed genes in WM266-4 and 501mel cells. Lists of transcriptional master regulators in the respective cell line compared to the other with a significant activation z-score are shown. Note that SMAD7, active in 501mel cells, inhibits TGFB signalling, where TGFB1, TGFB2, TGFB3, SMAD3 and SMAD4 indicate activated TGFB signalling. FGF2 and EGF are potent up-stream regulators of the MAPK pathway and hence AP1.

(b) Functional characteristics of WM266-4 and 501mel cells revealed by GSEA using the MSigDB hallmark gene set collection and the Hoek and Verfallie invasive and proliferative signatures (Verfaillie et al., 2015; Widmer et al., 2012).

(c) GSEA plots of the Hoek and Verfallie invasive signatures for 501mel and WM266-4 cells. Images of 501mel-mcherry and WM266-4-GFP spheroids embedded in collagen for 72h are shown.

(d) GSEA plots of the Hoek and Verfallie proliferative signatures for 501mel and WM266-4 cells. Relative cell number change over time for 501mel-mcherry and WM266-4-GFP cells is shown.


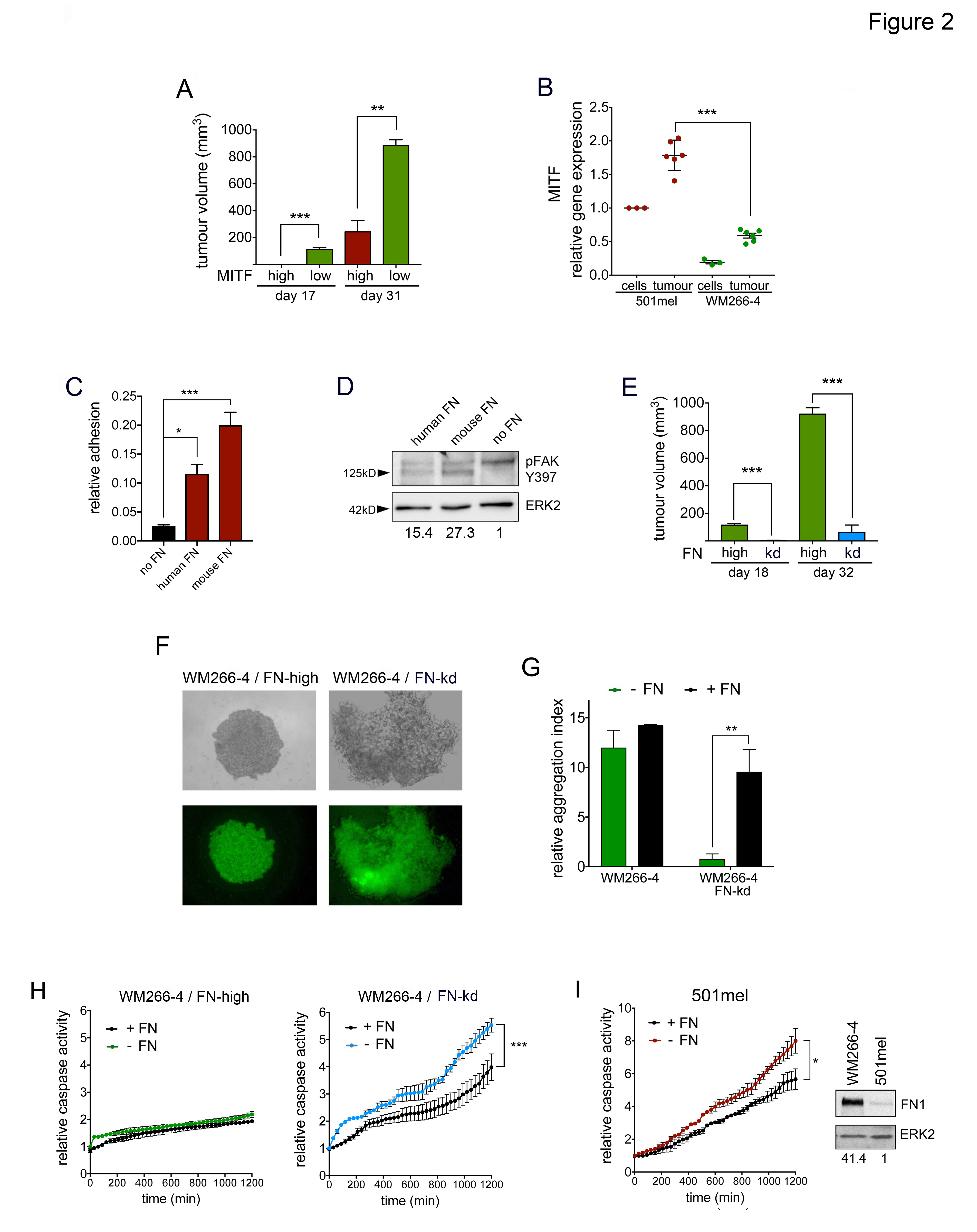


**Figure S2, related to Fig 1: High FN1 expression accelerates tumour growth**

(a) Tumour volumes at day 17 and 31 in mice (n = 3 mice/group) injected with 501mel-mCherry or WM266-4-GFP cells.

(b) qRT-PCR analysis of MITF expression in 501mel-mCherry compared to WM266-4-GFP cells and in corresponding tumours.

(c) Relative adhesion of 501mel cells to either human or mouse fibronectin or in the absence of fibronectin.

(d) Western blot analysis for phospho FAKY397 in 501mel cells adhered to human or mouse fibronectin or in the absence of fibronectin. Quantification of the relative intensity of p-FAK using ERK2 as reference is indicated.

(e) Tumour volumes at day 18 and 32 in mice (n = 4 mice/group) injected with WM266-4-GFP or WM266-4 FN-kd-GFP cells.

(f) Brightfield and GFP-fluorescence images of melanoma spheres formed by WM266-4-GFP and WM266-4 FN-kd-GFP cells 10h after plating.

(g) Relative aggregation index related to (D) in the absence and presence of human plasma-derived fibronectin (FN).

(h) Relative NucView® 488-caspase activity over time was measured in aggregated WM266-4-GFP or WM266-4 FN-kd-GFP cells in the absence and presence of human plasma-derived fibronectin (FN) using the Incucyte imaging system.

(i) Relative caspase activity over time in 501mel-mCherry cells in the absence and presence of human plasma-derived fibronectin (FN). FN1 expression in 501mel and WM266-4 cells is shown, and quantification of the average relative intensity from four independent Western blots using ERK2 as reference is indicated.

Where indicated, values are the mean ± SEM. *p < 0.05; **p < 0.01; ***p < 0.001


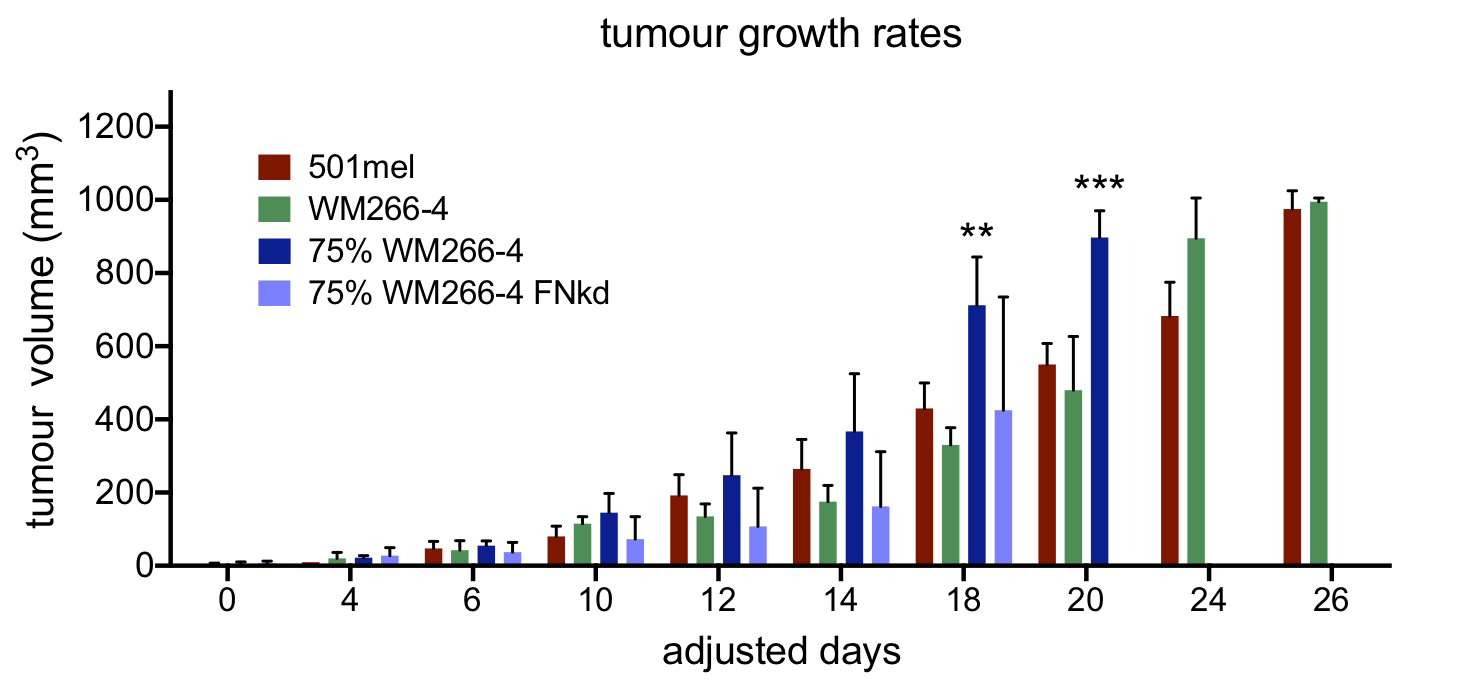


**Figure S3, related to Fig 3: Co-operativity accelerates tumour growth**

Adjusted tumour volumes over time in mice (n = 4 mice/group) injected with 501mel-mCherry or WM266-4-GFP cells either alone or in combination (75% WM266-4/25% 501mel). Tumour onset for each group has been set 0. Values are the mean ± SEM. **p < 0.01; ***p < 0.001


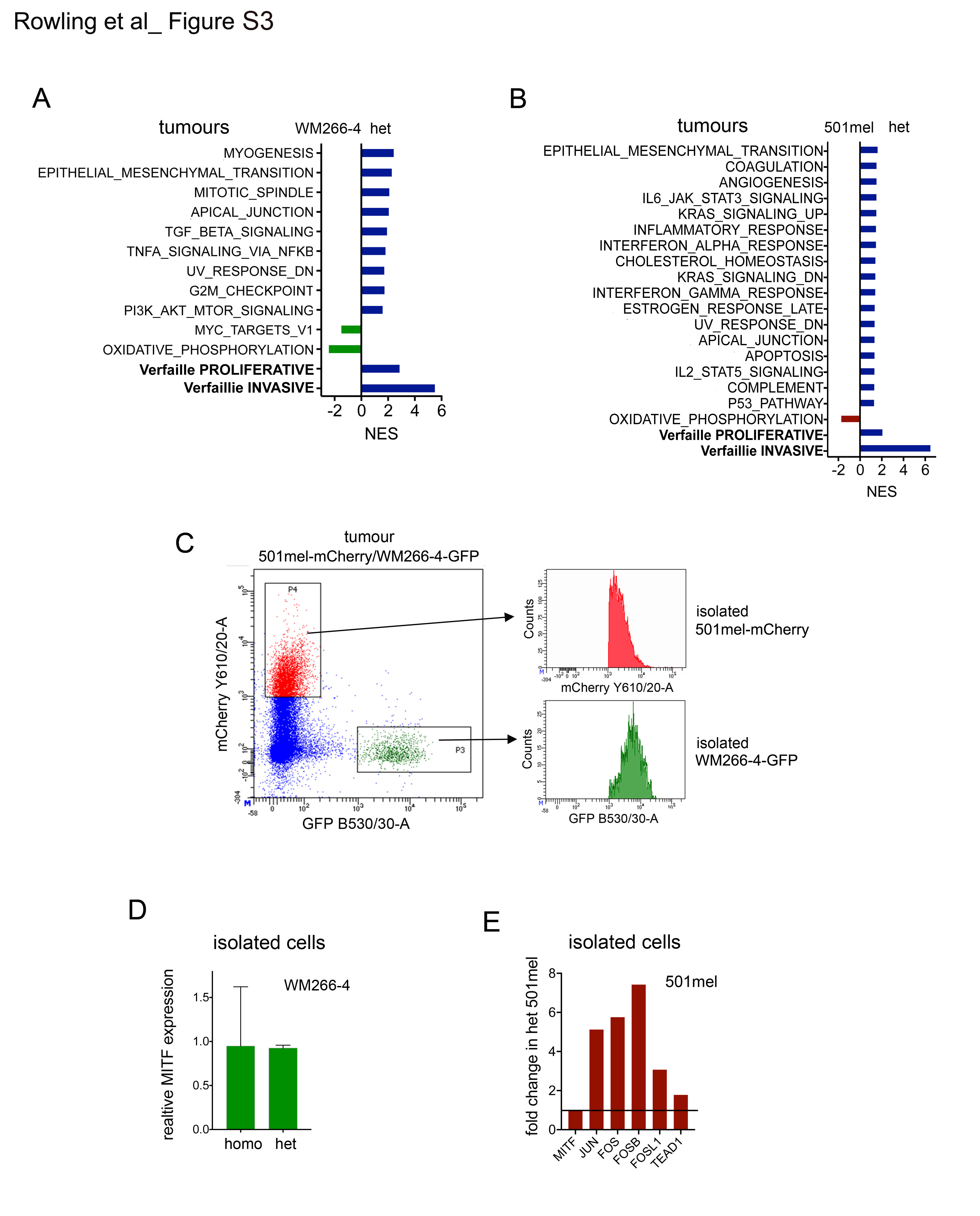


**Figure S4, related to Fig 4: Characterisation of heterogeneous tumours**

(a) Functional characteristics of WM266-4 tumours compared to heterogeneous tumours revealed by GSEA using the MSigDB hallmark gene set collection and the Verfallie invasive and proliferative signatures.

(b) Functional characteristics of 501mel tumours compared to heterogeneous tumours revealed by GSEA using the signatures as in (a).

(c) Isolation of 501mel-mCherry and WM266-4-GFP cells from heterogeneous tumours. Gating for GFP/mCherry was used to select GFP or mCherry positive cells and an RFP or GFP/Count graph was used to better observe the sorted sample.

(d) qRT-PCR for MITF expression in GFP-WM266-4 cells sorted either from homogeneous WM266-4 tumours or from heterogeneous tumours.

(e) Fold change in RNA-seq expression data (cut-off p < 0.05) for the indicated genes in 501mel-mCherry cells sorted from heterogeneous compared to homogeneous tumours.


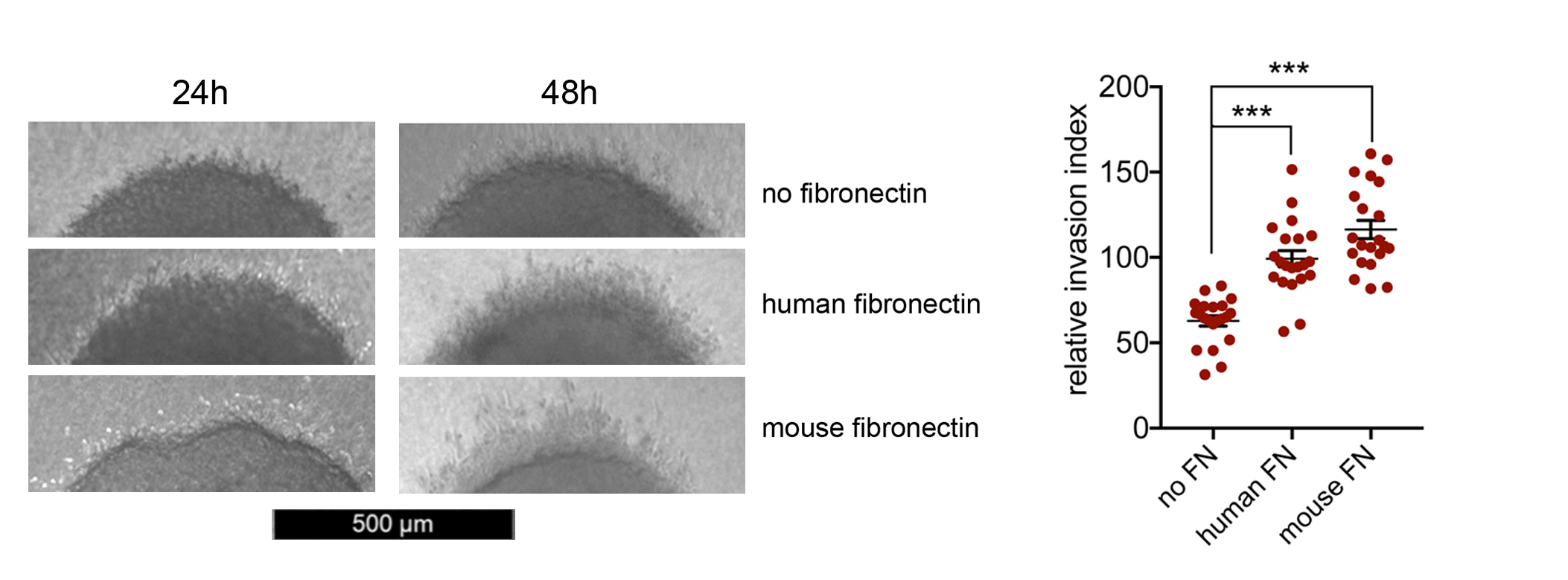


**Figure S5, related to Fig 5: Mouse fibronectin aids 501mel cell invasion**

Relative invasion of 501mel cell spheroids embedded in 3D collagen in either the absence or presence of human or mouse fibronectin. Images of invading cells at 24h and 48h after embedding are shown. The distance of invaded cells from the edge of the spheroid relative to the spheroid area was quantified using ImageJ. Indicated are 7 values each for n =3 spheroids per condition. Values are the mean ± SEM. ***p < 0.001

## Supplemental Information Materials and Methods

**Animal imaging procedure and analysis**

For stable luciferase expression, cells were transfected with MSCV-IRES-Luciferase-GFP (Zuber et al, 2009). Animals were imaged using the Bruker *In Vivo* Xtreme imaging system (Bruker Ltd, Coventry, UK) to measure disease burden. Eight minutes prior to imaging, animals were injected i.p. with 150mg/kg of luciferin (5ml/kg). Mice to be imaged were then anaesthetised with 2% isoflurane with 2L/min O_2_, transferred to the imaging bed and maintained at a surgical level of anaesthesia throughout the procedure. Animals were ventrally imaged for bioluminescence as per the methodology described in the Bruker *In Vivo* Xtreme operator’s manual, using Bruker MI software version 7.5.2.22464 for data acquisition and analysis. All parameters were kept identical. After the exposure an X-ray picture was taken to aid orientation and organ positioning. When imaging was complete, each animal was removed from the anaesthetic rig and returned to its cage. At time of analysis, the images were converted to photons/second/mm^2^ (P/s/mm^2^), the scale was adjusted and the bioluminescence image was superimposed upon the X-ray image. A region of interest (ROI) was set to measure the bioluminescence signal from the whole animal. The size of the ROI (interior area of 157170 pixels) was kept identical for all images, ensuring that the whole animal was contained within this area.

**Isolation of cells from mice**

For isolation of cells from whole tumours, the tumours were cross chopped with a scalpel as small as possible and then dissociated with pre-warmed Liberase (Roche) at 50ug/ml in serum free media in a humidified incubator at 37^o^C for 120mins. Serum containing media was then added, to neutralise the enzymes, and the samples were centrifuged and re-suspended in PBS for FACS sorting.

Blood was removed by cardiac puncture while mice were under anesthesia. The blood was then diluted in PBS and separated by density gradient centrifugation using Ficoll-Paque (GE Healthcare). The plasma and PBMC layer was then carefully removed and diluted in PBS for FACS sorting. For quantification, the plasma and PBMC layer was diluted in media and plated into a 6 well plate topped up with media containing pen/strep. Plates were then incubated and left until tangible colonies were visible. These colonies were them imaged using a fluorescent microscope and then fixed in ethanol and stained with methylene blue solution (Fisher scientific) for counting.

**Sorting of mCherry-501mel and GFP-WM2664 cells**

Cell sorting from tumours was performed using a BD Biosciences FACS Aria Fusion with the excitation laser 488nm and the emission filter 530/30nm bandpass for GFP and excitation laser 561nm and the emission filter 610/20nm bandpass for mCherry. The gating was set as follows: FCS-H/FSC-A was used to exclude any events that could represent more than 1 cell and FSC/SSC were used to gate out debris based on size. GFP/mCHerry was used to select GFP or mCherry positive cells (Figure S4c). Then an RFP or GFP/Count graph was used to better observe the sorted sample (Figure S4c). Immediately after FACS sorting samples were centrifuged, PBS removed and the cells were thoroughly resuspended in Qiazol. RNA was extracted using an Rneasy Plus Micro Kit (Qiagen).

**RNAseq experiment and data analysis**

Total RNA was isolated from 1-3 biological replicates per sample and submitted to the Genomic Technologies Core Facility (GTCF), University of Manchester. Quality and integrity of the RNA samples were assessed using a 2200 TapeStation (Agilent Technologies) and then libraries generated using the TruSeq® Stranded mRNA assay (Illumina, Inc.) according to the manufacturer’s protocol. Briefly, total RNA (0.1-4ug) was used as input material from which polyadenylated mRNA was purified using poly-T, oligo-attached, magnetic beads. The mRNA was then fragmented using divalent cations under elevated temperature and then reverse transcribed into first strand cDNA using random primers. Second strand cDNA was then synthesised using DNA Polymerase I and RNase H. Following a single 'A' base addition, adapters were ligated to the cDNA fragments, and the products then purified and enriched by PCR to create the final cDNA library. Adapter indices were used to multiplex libraries, which were pooled prior to cluster generation using a cBot instrument. The loaded flow-cell was then paired-end sequenced (76 + 76 cycles, plus indices) on an Illumina HiSeq4000 instrument. Finally, the output data was demultiplexed (allowing one mismatch) and BCL-to-Fastq conversion performed using Illumina’s bcl2fastq software, version 2.17.1.14

The Fastq files generated were analysed with FastQC (Andrews, S, Babraham Bioinformatics group, <http://www.bioinformatics.babraham.ac.uk/projects/fastqc/>) and any low quality reads and contaminated barcodes are trimmed with Trimmomactic (Bolger et al., 2014). All libraries were aligned to GRCh38.p12.genome assembly of human genome using STAR-2.5.3a (Dobin et al., 2013) and only the uniquely alighmened reads are used in the count step. The mapped reads are counted with HTSeq (Anders et al., 2015) at gene level agaist gencode.v28.annotation.gtf. R is used for all the statistics of the data analysis (R core Team, 2018). The counts data was normalised by geomitric means (Love et al., 2014). Differentially expressed genes are detected with a R package of DESeq2 (Love et al., 2014) between groups of experimental samples. Gene set enrichment analysis is carried out by using the Molecular Signatures Database Hallmark Gene Set Collection (Liberzon et al., 2015) from the MsigDb of the Broad Institute (Subramanian et al., 2005) and the enrichment test was carried out by using the pre-ranked gene list from DESeq2 with a fasta enrichment R package of fgsea (Sergushichev, A, 2016). The cluster analysis is carried out on the DE genes identified with DESeq2 using a padj cutoff of 0.05 with a dist and hclust (R core Team, 2018) and gplots (Warnes et al., 2016).

**RNA isolation for qPCR analysis**

Cell pellets of single 501mel and WM266-4 cells were lysed with Qiazol (Qiagen). Pieces of tumour, liver and lung were homogenized in Qiazol using a Precellys (Bertin Instruments). RNA was DNase treated (Qiagen) and reverse transcription performed using Omniscript (Qiagen), dNTPs (Qiagen), RNase inhibitor (Biolabs) and random hexamers (Applied Biosystems). RT-PCR was performed using SYBR Green Jumpstart (Sigma) and a Chromo4 qPCR system (BioRad) with triplicate biological repeats for each sample, and fold change calculated normalised to beta-actin expression. Primers were as follows: M-MITF: CCGTCTCTCACTGGATTGGT, TACTTGGTGGGGTTT-TCGAG TYROSINASE: ctggaaggatttgctagtccac, cctgtacctggga­cattgttc; MLANA: TTGGCACTCAATGTCCTTA, GGGAACCAC­AGGTTCACAGT ; TRPM1: ACCCAGAGCTACCCAACAGA, GTGAGTCTGGCTTGGTGTCA; CDK2: ATGGAGAACTTCCAAAAGGTGGA, CAGGCGGATTTTCTTAAGCG; CDH2: CATCCAGACCGACCCAAACA, GGCACTTGATTTTCTGCAGC; Human FN1 CTGG-CCAGTCCTACAACCAG, GGAATCTTCTCTGTCAGCCTGT; b-Actin: Gcaagcag-gagtatgacgag, Caaataaagccatgccaatc; for TaqMan qPCR the individual probes were: ACTB:Hs99999903; CDH1 QT00080143 Mouse FN1 QT00135758.

**IHC and Immunofluorescence staining of tissue**

Formalin fixed, paraffin embedded samples were stained by immunohistochemistry. Briefly, after deparaffinisation, slides were treated with hydrogen peroxide, to inactivate endogenous peroxidases, and then boiled in citrate buffer for antigen retrieval. After incubation with primary antibodies against Fibronectin (F3648 Sigma) at 1:1000 or aSMA (18-0106 Invitrogen) at 1:200, a secondary antibody was used to detect protein expression. Finally, DAB was used for visualization followed by counterstaining with Hematoxylin. Frozen sections were permeabilised in 0.25% Triton-X (in PBS), blocked in 10% goat serum and incubated with Fibronectin (F3648 Sigma) at 1:500 overnight at 4^o^C. Alexa 350 Goat-anti Mouse (ThermoFisher) secondary antibody was then added to the slides followed by mounting and imaging using a fluorescent microscope.

**Spheroid aggregation and apoptosis assay**

For aggregation studies 5000 cells were resuspended in DMEM containing 5% FBS with or without 25ug/ml Fibronectin from Human Plasma (Sigma, UK) and were transferred into a 96 U-well plate. The plate was then imaged over 24 hours using an Incucyte imaging system (Essen BioScience) and the spheroid size and time taken for maximum aggregation was used to calculate the aggregation index. For apoptosis plates were set up as above and NucView® 488 Caspase-3 Assay Kit (biotium) was used to measure caspase activity over time using the Incucyte imaging system.

**Spheroid invasion assay**

501mel spheroids were generated using the hanging-drop method, and after formation, spheroids were collected and embedded in collagen (2.3 mg/ml, Nutacon, Netherlands) as previously described (Ferguson et al, 2013). Human (Sigma, UK) or mouse (Abcam, UK) fibronectin was added at a concentration of 25 µg/ml to the collagen mix before solidification.

**Cell-cell and cell-substrate adhesion assays**

Cells were plated in 96 well plates and left until 90% confluent. The appropriate cells were then counted and 10,000 were resuspended and incubated at 37^o^C in solution for 30mins for adhesion molecule turnover. Media was then removed from the 96 well plate and the cell suspension added and incubated for 30-300 minutes. Cells were then washed, fixed with formalin and imaged using a fluorescent microscope.

For the assessment of direct adhesion of 501mel cells to human or mouse fibroenctin, 96 well plates were coated with 5µg/ml of the respective fibronectin or left uncoated. Cells in suspension were added to the plate and allowed to adhere to the substrate for 60min. Non-adhering cells were washed off with phosphate-buffered saline, and adhering cells were formalin fixed, stained with 0.5% toluidine blue, and the solubilised colour (in 1% sodium dodecyl sulphate) was measured at 595 nm.

**Cell lysis and immunoblotting**

Cells were lysed in SDS sample buffer, were separated by SDS-PAGE and transferred to nitrocellulose membrane (ImmobilonP) according to standard protocols. Primary antibodies were detected and visualised using an anti-rabbit or anti-mouse horse radish peroxidase (HRP)-tagged secondary antibody (1:5000, GE Healthcare) and by the addition of chemi-luminescence substrate (ECL, Perkin-Elmer) and autoradiography, imaged using a ChemiDoc imaging system (Biorad). The primary antibodies used were Fibronectin (F3648 Sigma) at 1:1000 and ERK2 (sc-154 Santa Cruz) at 1:500.

**EdU Incorporation assay**

A Click-iT® EdU Alexa Fluor imaging kit (Thermo Fisher Scientific) was used to measure EdU incorporation. Briefly, subconfluent cells were incubated with 20uM EdU for 4 hours. Cells were then fixed, permeabilised and incubated with the Click-IT reaction mixture followed by counterstaining with DAPI and mounting with Vectashield (Vector Laboratories). Cells were imaged with a fluorescence microscope and EU incorporation into the nucleus was counted.

**Anoikis assay**

3x10^5^ cells suspended in low serum media were added to 6 well plates coated in 3% agarose (sigma) and incubated for 24, 48 or 72 hours. Cell survival was then measured by either using a NucView® 488 Caspase-3 Assay Kit (biotium) or by counting cells using trypan blue (Sigma) to exclude dead cells, and measuring GFP and mCherry levels using a Gen5 microplate reader (BioTek).

References

Anders, S., Pyl, P.T., and Huber, W. (2015). HTSeq--a Python framework to work with high-throughput sequencing data. Bioinformatics *31*, 166-169.

Bolger, A.M., Lohse, M., and Usadel, B. (2014). Trimmomatic: a flexible trimmer for Illumina sequence data. Bioinformatics *30*, 2114-2120.

Dobin, A., Davis, C.A., Schlesinger, F., Drenkow, J., Zaleski, C., Jha, S., Batut, P., Chaisson, M., and Gingeras, T.R. (2013). STAR: ultrafast universal RNA-seq aligner. Bioinformatics *29*, 15-21.

Ferguson, J., Arozarena, I., Ehrhardt, M. & Wellbrock, C. (2013) Combination of MEK and SRC inhibition suppresses melanoma cell growth and invasion, Oncogene. 32, 86-96.

Liberzon, A., Birger, C., Thorvaldsdottir, H., Ghandi, M., Mesirov, J.P., and Tamayo, P. (2015). The Molecular Signatures Database (MSigDB) hallmark gene set collection. Cell Syst *1*, 417-425.

Love, M.I., Huber, W., and Anders, S. (2014). Moderated estimation of fold change and dispersion for RNA-seq data with DESeq2. Genome Biol *15*, 550.

R Core Team (2018). R: A language and environment for statistical computing. R Foundation for Statistical Computing, Vienna, Austria. URL [https://www.R-project.org/](https://www.r-project.org/).

Sergushichev, A. (2016) An algorithm for fast pre-ranked gene set enrichment analysis using cumulative statistic calculation. BioRxiv, doi:10.1101/060012

Subramanian, A., Tamayo, P., Mootha, V.K., Mukherjee, S., Ebert, B.L., Gillette, M.A., Paulovich, A., Pomeroy, S.L., Golub, T.R., Lander, E.S.*, et al.* (2005). Gene set enrichment analysis: a knowledge-based approach for interpreting genome-wide expression profiles. Proc Natl Acad Sci U S A *102*, 15545-15550.

Verfaillie, A., Imrichova, H., Atak, Z.K., Dewaele, M., Rambow, F., Hulselmans, G., Christiaens, V., Svetlichnyy, D., Luciani, F., Van den Mooter, L.*, et al.* (2015). Decoding the regulatory landscape of melanoma reveals TEADS as regulators of the invasive cell state. Nat Commun *6*, 6683.

Warnes, GR, Bolker, B, Bonebakker, L, Gentleman, R, Huber, W, Liaw, A, Lumley, T, Maechler, M, Magnusson, A, Moeller, S, Schwartz, M and Venables, B (2016). gplots: Various R Programming Tools for Plotting Data. R package version 3.0.1. [https://CRAN.R-project.org/package=gplots](https://cran.r-project.org/package=gplots)

Widmer, D.S., Cheng, P.F., Eichhoff, O.M., Belloni, B.C., Zipser, M.C., Schlegel, N.C., Javelaud, D., Mauviel, A., Dummer, R., and Hoek, K.S. (2012). Systematic classification of melanoma cells by phenotype-specific gene expression mapping. Pigment Cell Melanoma Res *25*, 343-353.

Zuber, J., Radtke, I., Pardee, T.S., Zhao, Z., Rappaport, A.R., Luo, W., McCurrach, M.E., Yang, M.M., Dolan, M.E., Kogan, S.C.*, et al.* (2009). Mouse models of human AML accurately predict chemotherapy response. Genes Dev *23*, 877-889.
